# Assessment of Post-Stroke Consequences in Pediatric Ischemic Stroke in the Context of Neuroimaging Results—Experience from a Single Medical Center

**DOI:** 10.3390/children8040292

**Published:** 2021-04-08

**Authors:** Ilona Kopyta, Beata Sarecka-Hujar, Dorota Raczkiewicz, Katarzyna Gruszczyńska, Magdalena Machnikowska-Sokołowska

**Affiliations:** 1 Department of Pediatric Neurology, Faculty of Medical Sciences in Katowice, Medical University of Silesia in Katowice, 40-752 Katowice, Poland; ilonakopyta@autograf.pl; 2 Department of Basic Biomedical Science, Faculty of Pharmaceutical Sciences in Sosnowiec, Medical University of Silesia in Katowice, 41-200 Sosnowiec, Poland; bsarecka-hujar@sum.edu.pl; 3 Department of Medical Statistics, School of Public Health, Center of Postgraduate Medical Education, 01-826 Warsaw, Poland; 4 Department of Diagnostic Imaging, Radiology and Nuclear Medicine, Faculty of Medical Sciences in Katowice, Medical University of Silesia in Katowice, 40-752 Katowice, Poland; kgruszczynska@poczta.onet.pl (K.G.); magdams@onet.pl (M.M.-S.)

**Keywords:** arterial ischemic stroke, outcome, children, neuroimaging, magnetic resonance imaging

## Abstract

Arterial ischemic stroke (AIS) in children is a rare condition; its frequency is estimated at 0.58 to 7.9 new onsets in 100,000 children per year. The knowledge on risk factors, clinical outcomes and consequences of pediatric AIS is increasing. However, there are still many unknowns in the field. The aim of the study was to analyze the clinical presentation of pediatric AIS and its consequences according to the neuroimaging results and location of ischemia. The research was retrospective and observational. The analyzed group consisted of 75 AIS children (32 girls, 43 boys), whereby the age of the patients ranged from 9 months to 18 years at stroke onset. All the patients were diagnosed and treated in one tertiary center. The most frequent stroke subtype was total anterior circulation infarct (TACI) with most common ischemic focus location in temporal lobe and vascular pathology in middle cerebral artery (MCA). The location of ischemic focus in the brain correlated with post-stroke outcomes: intellectual delay and epilepsy, hemiparesis corresponded to the location of vascular pathology. A correlation found between ischemic lesion location and vascular pathology with post-stroke consequences in pediatric AIS may be important information and helpful in choosing proper early therapy. The expected results should lead to lesser severity of late post-stroke outcomes.

## 1. Introduction

Arterial ischemic stroke (AIS) is characterized by sudden onset of focal or generalized brain function disturbances caused by cerebral vascular dysfunction and correlated with the location of vascular pathology; the symptoms have to last for over 24 h [1].

The up-to-date literature provides many studies on epidemiology, clinical presentation, risk factors, treatment and prevention of AIS in adults. In the last few years the research on AIS in pediatric populations has become more frequent—in literature reviews many former manuscripts were based on small patient cohorts. The presentation of ischemic lesions in the results of neuroimaging studies is, in some cases, inconsistent with the clinical presentation. This happens, for example, in patients with sickle cell disease (SCD) in whom new stroke lesions visible in an magnetic resonance (MR) of the brain do not show clinical symptoms and such a condition is called a silent stroke [2].

The occurrence of strokes in children, including arterial ischemic stroke, is commonly defined as a rare condition. There are not many papers on AIS prevalence in pediatric populations. The additional problem is the age of children enrolled in research on AIS. Newborns are usually excluded from pediatric stroke research; stroke in children below the first month of life is called neonatal stroke and its occurrence is higher compared to older children, whereby the risk factors for neonatal strokes are age-specific. Similarly, the upper limit of the age of children with stroke in research papers, differs between 16 to 20 years. This causes differences in the determination of AIS occurrence in the pediatric population. The frequency of stroke subtypes, including AIS, intracranial hemorrhage (ICH), or subarachnoid hemorrhage (SAH) may differ between the described patient groups [3,4]. In the study by Schoenberg et al. [4], the incidence of AIS in children up to the age of 15 years was established at 0.6 new cases per 100,000 children per year. In comparison, studies by Zahuranec et al. [3] and by Laugesaar et al. [5], performed at the beginning of the 21st century, indicated a higher frequency of AIS (1.1 and 1.6/100,000 children per year, respectively).

In most cases of AIS, the differentiation between primary hemorrhagic stroke and hemorrhagic transformation of primary ischemic focus is not problematic. However, in some specific situations, mostly in a large primary ischemic lesions, it may not be obvious. This may lead to misclassification of patients and inclusion AIS cases to groups a hemorrhagic stroke, and finally to contribute to different results of the studies on frequency of both ischemic and hemorrhagic strokes [6]. 

The etiology of childhood AIS is complex and multifactorial, often with multiple risk factors diagnosed in one patient. The groups of risk factors for childhood stroke involve: cerebral arteriopathies, congenital and acquired heart diseases, congenital and acquired conditions conducive to thrombosis, as well as injuries and intoxications. Congenital heart defects and acquired cardiovascular diseases may be considered as independent causes of an AIS in children, while in 30% of children, secondary cerebral ischemia develops during procedures related to the treatment of these conditions [7]. AIS may also occur in children after cardiac catheterization [8].

Cerebral arteriopathy is a large group of risk factors for childhood stroke, with the focal cerebral arteriopathy of childhood (FCA) and cranio-cervical arterial dissection (CCAD) as the most common ones. Imaging diagnostics using non-invasive magnetic resonance imaging (MRI) methods allow for the correct diagnosis and treatment in these cases, avoiding invasive procedures [9]. In turn, SCD is a very specific problem in terms of the occurrence of childhood strokes and their recurrences, as well as the attack of the “silent stroke”. Due to the high prevalence of SCD, e.g., in the Mediterranean region, the wide literature data are devoted to this issue; however, treating patients remains a challenge [10].

Ischemic stroke most commonly concerns children at pre-school and early school-age (5th–9th year of life), being slightly more often recognized in boys than girls [3,11,12].

Clinical presentation of stroke depends on the location of vascular pathology and the site of ischemic focus. Circulation disorders within the anterior part of the brain vasculature, i.e., internal carotid artery (ICA) in its intracranial part, as well as MCA, cause hemiparesis. Less commonly, it causes paralysis of the extremities, ipsilateral central facial nerve palsy and contralateral to the location of ischemic focus, hemianopia, consciousness and speech disturbances (motor and/or sensory aphasia), if the stroke concerns the dominant brain hemisphere. Occasionally, the symptoms listed above are accompanied or preceded by headache, nausea, vomiting and/or convulsions, which represent symptoms of intracranial increased pressure (ICP). The symptoms occurring commonly in newborns, like consciousness disturbances and seizures, are non-specific and not strictly bound to ischemic focus location. When circulation disturbances concern the posterior vascularization circle, the features of cerebellar syndrome will dominate the clinical presentation. The commonly used classification of AIS is based on the location of vascular pathology (Table 1) [13,14,15].

Immaturity of the central nervous system, multiple etiological factors and causes of pediatric stroke and unspecific clinical signs, which often mimic other diseases, lead to difficulties in differential diagnosis, which is not possible without imaging. Sensitivity of patients in the developmental age leads us to choose low radiation methods. Additionally, computed tomography (CT) is very often not sensitive enough in an acute phase of stroke in children to make a certain diagnosis. This makes MRI a crucial diagnostic method for pediatric stroke. Using short protocols, specifically the assessment of water diffusion disturbances, can give quick and precise information on anatomic association with clinical signs. Additional use of angioMRI brings us closer to the link between possible vascular pathology and the morphologic location of stroke. Unfortunately, angioMRI of Willis circle may sometimes not be sufficient for vessel evaluation. At this point, angioCT, having better spatial resolution, would be the method of choice. All of these factors might affect treatment options depending on the possible etiology of stroke [16,17,18]. Imaging is a key for diagnosis in suspected pediatric stroke, and MRI, with the benefit of sensitivity and no radiation, becomes the method of choice. Previously, Fullerton et al. [19] observed that cerebrovascular imaging identifies pediatric patients at the highest risk for recurrence in late childhood. They observed no recurrences among children with normal vascular imaging. However, children with a vascular abnormality had a 5-year cumulative recurrence rate of 66% [19].

The aim of the present study was to analyze the post-stroke consequences in the group of pediatric patients suffering from their first AIS, treated in a single medical center in southern Poland, in the context of neuroimaging results.

## 2. Materials and Methods

### 2.1. Study Group

The study was retrospective and was performed on the AIS pediatric patients’ medical records. We found the records of AIS patients’ by ICD-10 (I63, I64, I68, G46) in the hospital record systems. Each record found in this way was manually checked thereafter. The analyzed group consisted of 75 children who suffered from the first ever AIS and in an acute phase of the disease. They were hospitalized at the Department of Pediatric Neurology, Medical University of Silesia in Katowice. The group included 32 girls and 43 boys. The diagnosis of AIS was based on the up-to-date criteria. The following inclusion criteria were adopted:

- Age of patients at AIS onset over 29th day of life up to the completion of the 18th year of life;
- Focal or generalized brain function disturbances lasting over 24 h;
- Statement of presence of ischemic focus/foci on neuroimaging—CT and/or MRI—performed in acute phase of disease, usually in 24–48 h of symptomatic onset.
- The exclusion criteria were as follows:
- Patients with neonatal stroke;
- Children diagnosed with transient ischemic attacks (TIA), craniocerebral injuries, neuroinfections and other problems being a cause of acute central nervous system damage and clinical symptoms;
- Patients with neuroimaging confirmation of hemorrhagic stroke and cerebral sinus venous thrombosis (CSVT).

The study was reported to the local bioethics committee, which issued a resolution that consent to the study is not required as it is retrospective and consists of analyzing patient documentation (KNW/0022/KB/88/18).

According to definitions used in adult populations, AIS patient imaging results may be evaluated in the following stroke stages: early hyperacute (0–6 h form symptoms onset), late hyperacute (6–12 h), acute (24 h to 1 week), subacute (1–3 weeks), and chronic (more than 3 weeks) [20].

### 2.2. Neuroimaging

In all patients, brain imaging was performed—CT in 55 patients and/or MRI in 63 patients. In the first stage of analysis only CT was accessible. As the paper has retrospective character and in the analyzed period of time the access to examinations by a MR method was limited, in some cases only CT was performed. The localization of vascular pathology was classified according to the major brain arteries: in MCA, anterior cerebral artery (ACA), ICA and posterior cerebral artery (PCA). Location of ischemic focus/foci was evaluated in all analyzed pediatric patients.

Results of neuroimaging were assessed by radiologists experienced in the diagnosis of pediatric stroke.

### 2.3. Computed Tomography Protocol

The CT examinations were performed with a 16-row scanner TOSHIBA Aquilion S 16 (TMS Toshiba Medical System Corporation, Otawara, Japan), using spiral technique, slice thickness 0.5 mm, 100 KV, with the diagnostic protocols adjusted to child age. The obtained CT axial images were analyzed using dedicated Toshiba workstation (3Dnet TM Medical HTML5 Platform ver. 2.15, Biotronics 3D Ltd., London, UK).

### 2.4. MRI Protocol

All subjects were scanned using a 1.5 T MR imaging system—Optima 450 w GEM (General Electric, GE Healthcare, Milwaukee, WI, USA). Imaging protocol included T1, T2, T2 Flair, SWI (susceptibility weighted image) and DWI (diffusion weighted image) sequences. MR angiography was performed using a TOF (time of flight) technique, which allows visualization of vessels without intravenous contrast injection. T1 3D post contrast sequences were performed in doubtful cases. The dose of gadolinium contrast was 0.1 mL/kg of body weight. DWI was the most important sequence in stroke patients as abnormalities in water diffusion in brain tissue change signals as early as 30 min after a stroke incident.

In the early hyperacute phase (first six hours after stroke) and with uncooperative patients, a fast protocol was applied with DWI, T2 Flair and T2 sequences.

Radiological limitations of movement artefacts—especially in the most valuable sequences—necessitated the use of quick sedation, even in some older children. All children up to six years old were sedated for examination.

### 2.5. Image Analysis

The data were transferred to a commercially available workstation. A 3D volume of spiral CT acquisition was reoriented into three planes in a multiplanar reconstruction technique (MPR). The same reconstruction was used for multiplanar assessment of 3D acquisitions in MRI. 3D TOF was processed into three planes in Maximum Intensity Projection (MIP) and into 3D angiography views. In every method, two radiologists conducted the analysis in consensus. DWI sequence was always compared to ADC maps (apparent diffusion coefficient), which differentiates between cytotoxic and vasogenic edema.

### 2.6. Neurological Examination at the AIS Onset and in the Follow-Up

Discerning interviews were taken in each patient, regarding the circumstances of AIS onset and the results of neurological examination in an acute phase of disease.

Each child with AIS underwent at least two detailed neurological examinations. On the basis of results of both neurological as well as neuroimaging examinations, the stroke was classified as one of four categories: most frequent were TACI and following this in terms of frequency were lacunar anterior circulation infarct (LACI), partial anterior circulation infarct (PACI), and posterior circulation infarct (POCI). Neurological examination during the follow-up aimed to evaluate the stroke consequences as follows: hemiparesis, aphasia (speech impairment), post-stroke epilepsy, intellectual delay as well as motor disturbances other than hemiparesis. Post-stroke epilepsy was defined as at least two recurrent, unprovoked seizures occurring after the acute phase of stroke. Intellectual delay and/or school difficulties were based on the observations and relation of the parents/caregivers of children with AIS. Motor impairments ranged from slight to medium intensity, according to our assumption.

### 2.7. Statistical Analysis

The data were statistically analyzed using Statistica 13.1 software (STATSOFT, Tulsa, OK, USA) as well as VassarStats software (R. Lowry, VassarStats: Website for Statistical Computation). Mean values (M) and standard deviations (SD) were estimated for continuous variables while absolute numbers (n) and relative numbers (%) were estimated for categorical variables. In order to compare vascular location between stroke subtypes, morphological changes location between stroke subtypes, post-stroke outcomes according to morphological changes locations as well as post-stroke outcomes according to vascular changes locations, the Fisher’s exact test along with the Freeman–Halton extension (for contingency tables greater than 2 × 2) was used. The significance level was set at *p* ≤ 0.05 in all statistical tests.

## 3. Results

### 3.1. Characteristics of the Patients

The general characteristics of children who underwent their first ever AIS according to sex, age (at stroke onset and at follow-up), stroke subtype, position of the child’s body and season of the year at onset of the disease is presented in Table 2.

In the analyzed group of patients with AIS over 57%, were boys. Mean age of stroke onset was 8.4 years. The youngest child was 9 months old at AIS onset, with the oldest being 18 years. The age of patients at follow-up was between 18 months and 26 years of age. The follow-up observation time was between 6 months and 11 years, with the mean 2 years and 10 months. The mean age of AIS patients at control examination was 13 years of age. The most common stroke subtype was TACI, which was present in 32% of children. Two children with TACI as a first stroke presented stroke recurrence with the second as POCI type. The least frequent stroke subtype was POCI, diagnosed in 13% of children. Stroke onset commonly occurred during child’s sleep (over 37% of the group), then during physical activity—mainly walking (28%); 20% of children were sitting at disease onset and less than 3% of them were standing during AIS onset.

The most typical season of the year for stroke onset was winter (in almost 35% of cases), the least, summer (in almost 19% of cases). Chronic diseases and congenital defects were found in 24 children of the group (32%). In seven children (9.33%), stroke onset occurred during 72 h after cardiac surgical procedure, in two (2.67%)—post adenotonsillectomy, in two (2.67%)—post Hirschprung disease surgical treatment, and in one child (1.33%) as the complication of varicella. In two children (2.67%), AIS was preceded by TIA in past history. A single focus was observed significantly more often compared to two or more foci (81% vs. 19%, respectively, *p* < 0.001, χ^2^ = 57.19). Two ischemic foci were observed in 11% of children with AIS, three foci in 5%, while multiple foci—in 3%. An accompanying brain edema was reported in 58% of children, hemorrhagic transformation in 23%. Vascular changes in the acute phase of disease, i.e., any pathology of the brain arterial vessels, were found in neuroimaging in 42 out of 75 analyzed children with AIS (56%). In an acute stage, full vascular patency was seen in 24%, narrowing of the vessel in the largest group (64%) and total occlusion in 12% of patients. Narrowed or occluded vessels were reported significantly more often compared to those totally patent (*p* < 0.001, χ^2^ = 27.04).

### 3.2. CT and MRI Reports

In 63 of the pediatric patients (84.0%), brain imaging was performed by MRI, while in 55 (73.33%)—by CT. In turn, in 43 children (57.33%) both MRI and CT were conducted. The results of neuroimaging were evaluated by pediatric radiologists experienced in childhood stroke.

**Figure 1:**
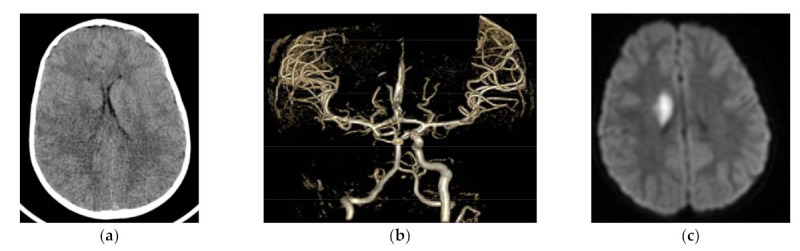
Acute focus of LACI (lacunar anterior circulation infarct) in right caudate nucleus: (**a**) CT (computed tomography) six hours after the onset—very subtle density blurring at the level of right caudate nucleus; (**b**) CTA (computed tomography angiography)—occlusion of right ICA (internal carotid artery) with sustained flow in MCA (middle cerebral artery) and ACA (anterior cerebral artery); (**c**) MRI DWI (diffusion weighted imaging in magnetic resonance)—restricted diffusion in right caudate nucleus region.

**Figure 2:**
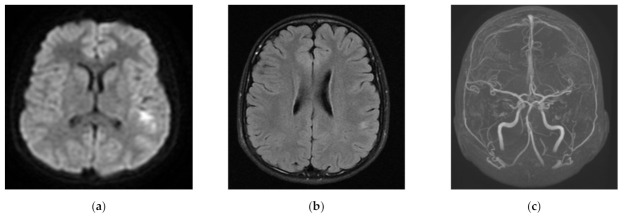
Discreet disseminated PACI (partial anterior circulation infarct) foci in left posterior temporal and parietal cortex/subcortical region: (**a**) MRI DWI (diffusion weighted imaging in magnetic resonance)—restricted diffusion in left posterior temporal/parietal cortex; (**b**) Flair MR—subtle signal changes in the same region; (**c**) MRA (magnetic resonance angiography)—occlusion of distal (cortical) branches of left MCA.

Table 3 demonstrates prevalence of morphological changes in the context of stroke subtypes. The most common morphological location of ischemic lesion was the temporal lobe, present in almost 74% of children with AIS. The majority of changes in the temporal lobe occurred in children with LACI stroke (91.30%), then in PACI and TACI stroke (83.33% and 75.00%, respectively). In the patients with LACI stroke, ischemic foci were limited mainly to temporal lobe only or both temporal and frontal lobes, present in 83% and 13%, respectively (Figure 1). Temporal lobe changes occurred in almost 84% with PACI subtype (Figure 2). Anatomic changes in the frontal lobe were found in over 33% of cases in parietal lobe, in 24% and in the posterior lobe, and in over 14% of patients in the total study group. Cerebellar changes were found in over 6% of children—they presented mostly as a POCI stroke. When we analyzed coexisting lesions (either 2, 3, or 4), significant differences were found in their prevalence according to stroke subtypes.

**Figure 3:**
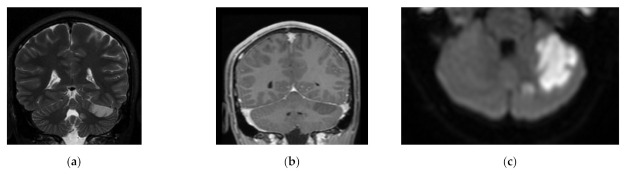
Focus of POCI (posterior circulation infarct) in the left SCA/AICA (superior cerebellar artery/anterior inferior cerebellar artery) territory in left cerebellar hemisphere in acute stage on the day of onset: (**a**) T2 MR; coronal plane; (**b**) T1 + CE coronal plane; (**c**) DWI MR—axial plane—restricted diffusion in the region.

Children with POCI presented the most frequent changes in the parietal lobe, cerebellum, pons, midbrain, and posterior lobe (Figure 3).

In the case of vascular changes, the most common location was observed in MCA—in over 57% of all AIS, and in ICA—in over 47% patients. Prevalence of vascular changes according to stroke subtypes is presented in Table 4. Less frequently, vascular changes were found in ACA and PCA (in over 26% and 14% of patients, respectively). The majority of vascular changes in MCA occurred in children with LACI stroke (71.43%) and next in TACI and PACI stroke (64.71% and 64.54%, respectively). None of the patients with POCI presented changes in MCA. In turn, vascular changes in ACA were found in over 41% of patients with TACI or in 40% of patients with POCI stroke while children with LACI had no changes in ACA. Analyzing 2, 3, or 4 co-existing vascular changes, significant differences were again observed in their prevalence according to stroke subtypes.

### 3.3. Post-Stroke Outcomes According to the Location of Vascular or Morphological Changes

Location of morphological changes correlated with intellectual delay and epilepsy, observed as post-stroke outcome (*p* = 0.015 and *p* = 0.054 on the border of significance) (Table 5). Among children with changes in the temporal lobe, 62% presented with hemiparesis in the follow-up. Post-stroke epilepsy and aphasia were observed with the same frequency (almost 9% each) in patients with changes in the temporal lobe. In turn, 6% of patients with temporal lobe changes presented movement disorders other than hemiparesis and 3%, intellectual delay (Table 6).

Presence of hemiparesis after AIS correlated with the location of vascular changes (*p* = 0.045) (Table 6). Among children with vascular changes in MCA, over 71% had hemiparesis after AIS, over 21% had post-stroke epilepsy and over 21% had aphasia. Over 28% of children with vascular changes in MCA presented an intellectual delay or school difficulties reported by parents or caregivers. Post-stroke movement disorders other than hemiparesis, mainly involuntary movements, were found in three children (over 7% of patients).

## 4. Discussion

AIS in children differs significantly in risk factors and etiology from AIS in adulthood. Clinical symptoms are similar, especially in older children, but alertness of primary care doctors is still is not sufficient. AIS in a child is seen as a very rare condition. Therefore, in primary care sometimes it is not considered as a potential cause of any acute symptoms observed; however, it is undoubtedly underestimated. Not only primary care clinicians but also radiologists do not have much experience in pediatric AIS diagnosis. This also contributes to the delay of the proper diagnosis and treatment. Currently, there are few reports on ischemic stroke in a given pediatric population or in a given geographic territory. In Poland, data based merely on low number of pediatric patients with AIS are available. There have been no population-based Polish surveys so far. Outcome and stroke consequences will be similar in children, young adults and elderly people. Their impact on the patient’s future life, however, differs significantly according to age. In children, the developmental delay caused by acute brain ischemia influences the final global achievements.

In our patients, epilepsy was most commonly seen in AIS children with an ischemic focus in the temporal lobe, while intellectual delay was mostly witnessed in patients with ischemia in the frontal lobe. In adults, the risk of epilepsy after AIS is up to 29% for TACI, 13% for PACI and 19–25% for cortical location of ischemic lesion. In experimental status epilepticus (SE) models, damage located in the subcortical or cortical temporal area drives the seizure-generating network [21]. Experimental data in animals on the influence of hypobaric hypoxia caused by living at a high altitude (even if the mechanism of brain ischemia is not of vascular origin in these cases), demonstrates that hypoxia is a reason for metabolic change in the frontal lobe and in this way may cause psychiatric disorders [22]. Few cases of adults after brain ischemia within the frontal lobe (confirmed by neuroimaging, with hypoperfusion on single photon emission tomography (SPECT)) show its role in specific psychiatric presentation with abulia [23].

The size of the study sample is comparable to those previously reported [24,25,26,27]. The gathered group is also homogenous in terms of origin and race—living in a rather small region of southern Poland. The number is further diminished by exclusion of hemorrhagic stroke patients, reported in almost 40% of all stroke patients. However, all patients were coming from one center and underwent the same study scheme in the meaning of the protocol of admission at hospital. This began from a family and medical history interview, through the physical and neurological examination, to neuroimaging in one imaging department. The results comprising a clinical picture of acute AIS and its consequences are similar to those published by other authors in the international literature [28].

The upper limit of the age of pediatric patients examined in our study group was 18 years old. The age of stroke patients analyzed in the context of pediatric AIS may be a problem, as neonates are excluded due to specific causes and an upper range is variable in different publications—from 15–16 years old [2,4], whereas in others, this is up to 20 years old [3]. These factors may also cause differences in reported pediatric AIS frequency.

In our study, a correlation was found between the presence of specific post-stroke outcome and location of vascular or morphological changes observed in neuroimaging. Clinical and anatomical changes were consistent with vascular location. Different approaches to small and large vessel disease etiology need to be worked upon, as invasive intravascular treatment of a large vessel—a standard option in adult stroke patients—is still in its early infancy in the pediatric population [17,18]. The standard procedure of neuroimaging in the population of pediatric stroke patients should be a brain MR supplemented with MR angiography. CT may be insufficient and pose additional problems of radiation [29]. Typical changes in vessels like hyperdense artery (seen in adults) are rare in children due to different stroke etiology. Assessment of pediatric brain stroke in CT is even more difficult because of the immaturity of the brain.

Unfortunately, in patients from early stages of our analysis, MRI was unavailable, so we were limited to CT. In our study, brain imaging by MRI was performed in the majority of patients, while almost 59% of analyzed patients started with CT as the first urgent method available. Whenever possible, CT was supplemented with MRI. Since we gained access to urgent 24/7 MRI examinations, all patients with infarct suspicion undergo MRI. As stated before, problems with pediatric MRI examination may be caused by an uncooperative patient—too young to lie still or with an altered consciousness. Claustrophobia poses additional problems, just like in adults. The shortest protocol including DWI and T2 and T2 FLAIR sequences last 15 min. MR angiography adds five minutes. Standard protocols including sequences as given in methodology last for 30 min. The main difficulties in MR imaging are caused by the time-consuming examination. Sedation is necessary in small children and uncooperative patients as described above.

Time-limiting factors in hyperacute (and any other stages) are rather connected to the cooperation of the patient and the eventual difficulties in sedation. The stage of the stroke is less important for protocol limitations.

In another study on Polish patients with AIS, CT was a primary diagnostic tool [30]. According to Bhatia and Pruthi, pediatric patients with suspected AIS should receive a complete evaluation of risk factors, and initial stroke imaging should include MRI and MRA of the brain [31]. The authors also recommend an MRA of the neck in the case of clinical suspicion for arterial dissection or cervical pathology, or in patients with unknown etiology of the disease [31]. In the study by Mallick et al. [32], AIS was initially diagnosed by CT in 66% of children, whereas MRI was diagnostic in 100%. According to the authors, MRI should be the initial imaging modality of choice in any suspected case of childhood AIS [32]. The updated diagnostic algorithm by Felling et al. [33] for children presenting with a new onset of AIS makes DWI in MRI the method of choice and, at the same time, MR angiography of the brain and neck should be performed. Performing CTA or DSA may be considered—if needed—thereafter. In turn, in retrospective research by Carey et al. [34], information on a head CT as an initial examination in all 158 children diagnosed in Manitoba, it was found that when CT scans were non-diagnostic, brain MRI or another CT was performed. In the adult AIS population considered for thrombectomy, the initial CT scan results evaluated by Combined Multimodal Computed Tomography Score (CMCTS) have not just a diagnostic value at the stroke onset, but also a present prognostic value to find the patients, who would not benefit from interventional treatment [35]. According to the up-to-date data by Donahue et al. [36] in the pediatric potential-stroke population, many conditions mimicking stroke, which would not be seen on CT scans must be taken into consideration. At this point, the additional radiation exposure on CT would be avoided in children. Up to 47% of ischemic changes may be missed in children if just CT is used, whereby the changes are properly diagnosed later with MR. Most younger children need general anesthesia for any neuroimaging, which is another reason to choose the method giving more chance to proper and fast diagnosis [36]. These recommendations are in opposition to the standard diagnostic protocol for adults with AIS, where CT and CTA are commonly the first line of imaging and the data are sufficient to decide about standard acute treatment.

Recent data have demonstrated that perfusion imaging is feasible in pediatric stroke, and may be helpful in identifying salvageable tissue in extended time windows [37].

The present study has some limitations. These include a low number of recruited patients, the upper age limit and also the ideal timing of examination. Children happened to be referred from different hospitals in the region, where the suspicion of stroke was not always evident at the onset. Therefore, initial imaging, even during the first hours after admission, was sometimes delayed up to 48 h after first symptoms. In previous data based on children with AIS from the UK, the median time from symptom onset of AIS to diagnostic neuroimaging was 24.3 h [32]. The authors also observed that if initial neuroimaging was non-diagnostic in AIS, then median time to diagnosis was 44 h [32]. Due to the retrospective character of the study and the fact that the diagnosis of AIS was done largely based on CT scan results, we have decided to use the Oxfordshire classification to distinguish the types of stroke. Currently, it is known that the evaluation simply based on clinical presentation and CT results differs from MR examination results, especially in the diagnosis of PACI stroke subtype [17]. Undoubtedly, an important weakness of our work is the fact that the mode of radiological evaluation was not unique for all patients, which resulted in a lack of full access to diagnostics using the MR method in some cases. When the CT image raised doubts by the radiologist as to the diagnosis of ischemic stroke, the MR examination was performed.

From a radiological point of view, further studies on vascular pathology, its evolution and correlation with risk factors, treatment options and complications should be closely evaluated. This could possibly be undertaken in a prospective study, which would be highly justified in the Polish population. The better our knowledge of pediatric stroke conditions, the greater the possibilities of both prevention and choice of treatment options we will have, including the education of patients and their families.

## 5. Conclusions

Modern neuroimaging procedures are crucial for proper and fast diagnosis in children with acute neurological symptoms. MRI is the preferred method due to its sensitivity in early stages, but can pose problems with uncooperative patients. Neuroimaging may be helpful in the prediction of post-stroke outcomes in children. According to own experience and up-to-date research on pediatric AIS, the method of choice should be MRI with a vascular option. This should be the purpose of pediatric AIS patient diagnosis improvement. Predicted long-term outcomes with hemiparesis, intellectual delay or epilepsy may push the care towards earlier and more specific rehabilitation of the patient and help with family cooperation. It is especially important to create diagnostic standards of AIS for children, as since late 2018, we can offer thrombolysis for the treatment of patients older than 16 years, thereby giving them a chance to avoid or diminish life-long stroke consequences.

## Figures and Tables

**Table 1:** AIS subtypes and their clinical presentation according to brain vasculature disturbance location [13,14,15].

AIS Subtype	Location	Clinical Presentation
PACI (Partial anterior circulation infarct)	Small or medium cortical stroke within MCA/ACA vasculature or large subcortical stroke within MCA	Hemiparesis/hemi paralysis contralateral to ischemic focus, aphasia, central facial nerve paresis
TACI (Total anterior circulation infarct)	Large or medium cortical stroke within MCA/ACA or large subcortical stroke within MCA	Consciousness disturbances, hemiparesis/hemi paralysis contralateral to ischemic stroke, aphasia, central facial nerve palsy
LACI (Lacunar anterior circulation infarct)	Small subcortical stroke within ICA	Hemiparesis, extrapyramidal symptoms
POCI (Posterior circulation infarct)	Stroke in brainstem or cerebellum	Ataxia, breathing disorders

AIS—Arterial ischemic stroke; MCA—Middle cerebral artery, ACA—Anterior cerebral artery, ICA—Internal carotid artery.

**Table 2:** General characteristics of children with AIS.

Characteristics, Parameter	Unit or Category	AIS Children (*N* = 75)
Gender (*n*, %)	Girls	32 (42.67)
	Boys	43 (57.33)
Age at AIS onset, min-max, (M ± SD)	Years	1–18 (8.4 ± 5.5)
Age at follow-up, min-max, (M ± SD)	Years	2–27 (12.1 ± 5.9)
Stroke subtype (*n*, %)	LACI	23 (30.67)
	TACI	24 (32.00)
	PACI	18 (24.00)
	POCI	10 (13.33)
Position of the body at AIS onset (*n*, %)	Sleeping	28 (37.33)
	Walking	21 (28.00)
	Sitting	15 (20.00)
	Standing	2 (2.67)
	no data	9 (12.00)
Season of the year at AIS onset (*n*, %)	Spring	16 (21.33)
	Summer	14 (18.67)
	Autumn	16 (21.33)
	Winter	26 (34.67)
	no data	3 (4.00)

AIS—arterial ischemic stroke; SD-standard deviation; LACI—lacunar anterior circulation infarct; TACI—total anterior circulation infarct; PACI—partial anterior circulation infarct; POCI—posterior circulation infarct, M—mean, SD—standard deviation.

**Table 3:** Prevalence of morphological changes locations according to stroke subtypes.

Prevalence of	Total (*N* = 75)	Stroke Subtypes				*p*
		LACI (*N* = 23)	PACI (*N* = 18)	TACI (*N* = 24)	POCI (*N* = 10)	
**Location of morphological changes, *n* (%)**						
Temporal lobe	55 (73.33)	21 (91.30)	15 (83.33)	18 (75.00)	1 (10.00)	**<0.001**
Pons	2 (2.67)	0 (0.00)	0 (0.00)	1 (4.17)	1 (10.00)	0.305
Cerebellum	5 (6.67)	0 (0.00)	0 (0.00)	2 (8.33)	3 (30.00)	**0.008**
Frontal lobe	25 (33.33)	3 (13.04)	8 (44.44)	12 (50.00)	2 (20.00)	**0.025**
Parietal lobe	18 (24.00)	0 (0.00)	7 (38.89)	9 (37.50)	2 (20.00)	**0.002**
Posterior lobe	11 (14.67)	1 (4.35)	0 (0.00)	8 (33.33)	2 (20.00)	**0.005**
Midbrain	1 (1.33)	0 (0.00)	0 (0.00)	0 (0.00)	1 (10.00)	0.133
**Coexisting morphological changes, *n* (%)**						**0.001**
Temporal lobe, Posterior lobe	3 (4.00)	0 (0.00)	0 (0.00)	2 (8.33)	1 (10.00)	
Temporal lobe, Parietal lobe	3 (4.00)	0 (0.00)	1 (5.56)	2 (8.33)	0 (0.00)	
Temporal lobe, Frontal lobe	6 (8.00)	3 (13.04)	2 (11.11)	1 (4.17)	0 (0.00)	
Frontal lobe, Parietal lobe	2 (2.67)	0 (0.00)	1 (5.56)	0 (0.00)	1 (10.00)	
Frontal lobe, Posterior lobe	1 (1.33)	0 (0.00)	0 (0.00)	1 (4.17)	0 (0.00)	
Temporal lobe, Frontal lobe, Posterior lobe	1 (1.33)	0 (0.00)	0 (0.00)	1 (4.17)	0 (0.00)	
Temporal lobe, Frontal lobe, Parietal lobe	7 (9.33)	0 (0.00)	4 (22.22)	3 (12.50)	0 (0.00)	
Temporal lobe, Pons, Cerebellum	1 (1.33)	0 (0.00)	0 (0.00)	1 (4.17)	0 (0.00)	
Frontal lobe, Posterior lobe, Parietal lobe	2 (2.67)	0 (0.00)	0 (0.00)	2 (8.33)	0 (0.00)	
Frontal lobe, Temporal lobe, Parietal lobe, Posterior lobe	1 (1.33)	0 (0.00)	0 (0.00)	1 (4.17)	0 (0.00)	
Frontal lobe, Parietal lobe, Posterior lobe, Cerebellum	1 (1.33)	0 (0.00)	0 (0.00)	1 (4.17)	0 (0.00)	
Temporal lobe only	34 (45.33)	19 (82.61)	8 (44.44)	7 (29.17)	0 (0.00)	
Pons only	1 (1.33)	0 (0.00)	0 (0.00)	0 (0.00)	1 (10.00)	
Cerebellum only	3 (4.00)	0 (0.00)	0 (0.00)	0 (0.00)	3 (30.00)	
Frontal lobe only	4 (5.33)	0 (0.00)	1 (5.56)	2 (8.33)	1 (10.00)	
Parietal lobe only	2 (2.67)	0 (0.00)	1 (5.56)	1 (4.17)	0 (0.00)	
Posterior lobe only	2 (2.67)	1 (4.35)	0 (0.00)	0 (0.00)	1 (10.00)	
Midbrain only	0 (0.00)	0 (0.00)	0 (0.00)	0 0.00()	1 (10.00)	

LACI—lacunar anterior circulation infarct; TACI—total anterior circulation infarct; PACI—partial anterior circulation infarct; POCI—posterior circulation infarct; *p* for Fisher’s exact text; Significant differences are in bold.

**Table 4:** Prevalence of vascular changes according to stroke subtypes.

Prevalence of	Total (*N* = 42)	Stroke Subtypes				*p*
		LACI (*N* = 7)	PACI (*N* = 13)	TACI (*N* = 17)	POCI (*N* = 5)	
**Location of vascular changes, *n* (%)**						
MCA	24 (57.14)	5 (71.43)	8 (61.54)	11 (64.71)	0 (0.00)	0.059
ACA	11 (26.19)	0 (0.00)	2 (15.38)	7 (41.18)	2 (40.00)	0.108
ICA	20 (47.62)	2 (28.57)	7 (53.85)	11 (64.71)	0 (0.00)	**0.050**
PCA	6 (14.29)	1 (14.29)	0 (0.00)	2 (11.76)	3 (60.00)	**0.017**
**Coexisting vascular changes, *n* (%)**						**0.022**
ICA, ACA	3 (7.14)	0 (0.00)	0 (0.00)	3 (17.65)	0 (0.00)	
ICA, MCA	6 (14.29)	1 (14.29)	3 (23.08)	2 (11.76)	0 (0.00)	
ICA, ACA, MCA	4 (9.52)	0 (0.00)	1 (7.69)	3 (17.65)	0 (0.00)	
ICA, MCA, PCA	1 (2.38)	0 (0.00)	0 (0.00)	1 (5.88)	0 (0.00)	
ICA, ACA, MCA, PCA	1 (2.38)	0 (0.00)	0 (0.00)	1 (5.88)	0 (0.00)	
MCA only	14 (33.33)	4 (57.14)	5 (38.46)	5 (29.41)	0 (0.00)	
ACA only	3 (7.14)	0 (0.00)	1 (7.69)	0 (0.00)	2 (40.00)	
ICA only	6 (14.29)	1 (14.29)	3 (23.08)	2 (11.76)	0 (0.00)	
PCA only	4 (9.52)	1 (14.29)	0 (0.00)	0 (0.00)	3 (60.00)	

LACI—lacunar anterior circulation infarct; TACI—total anterior circulation infarct; PACI—partial anterior circulation infarct; POCI—posterior circulation infarct; ICA—internal carotid artery; MCA—middle cerebral artery; ACA—anterior cerebral artery; PCA—posterior cerebral artery; *p* for Fisher’s exact text; Significant differences are in bold.

**Table 5:** Prevalence of post-stroke outcome according to location of morphological changes.

Location of Morphological Changes	Post-Stroke Outcome				
	Hemiparesis	Epilepsy	Aphasia	Movement Disorders Other than Hemiparesis	Intellectual Delay
	*n* (%)	*n* (%)	*n* (%)	*n* (%)	*n* (%)
Total (*N* = 75)	51 (68.00)	11 (14.67)	9 (12.00)	8 (10.67)	9 (12.00)
Temporal lobe only (*N* = 34)	21 (61.76)	3 (8.82)	3 (8.82)	2 (5.88)	1 (2.94)
Pons only (*N* = 1)	1 (100.00)	0 (0.00)	0 (0.00)	0 (0.00)	0 (0.00)
Frontal lobe only (*N* = 4)	3 (75.00)	0 (0.00)	2 (50.00)	0 (0.00)	2 (50.00)
Parietal lobe only (*N* = 2)	1 (50.00)	1 (50.00)	0 (0.00)	1 (50.00)	0 (0.00)
Posterior lobe only (*N* = 2)	0 (0.00)	0 (0.00)	0 (0.00)	0 (0.00)	0 (0.00)
Midbrain only (*N* = 1)	1 (100.00)	0 (0.00)	1 (100.00)	0 (0.00)	0 (0.00)
Cerebellum only (*N* = 3)	1 (33.33)	0 (0.00)	0 (0.00)	1 (33.33)	0 (0.00)
Frontal lobe, Parietal lobe (*N* = 2)	2 (100.00)	0 (0.00)	0 (0.00)	0 (0.00)	0 (0.00)
Frontal lobe, Parietal lobe, Posterior lobe, Cerebellum (*N* = 1)	1 (100.00)	1 (100.00)	1 (100.00)	0 (0.00)	1 (100.00)
Frontal lobe, Temporal lobe, Parietal lobe (*N* = 7)	6 (85.71)	2 (28.57)	1 (14.29)	2 (28.57)	2 (28.57)
Parietal lobe, Posterior lobe, Frontal lobe (*N* = 2)	2 (100.00)	0 (0.00)	0 (0.00)	0 (0.00)	0 (0.00)
Temporal lobe, Parietal lobe (*N* = 3)	3 (100.00)	2 (66.67)	0 (0.00)	0 (0.00)	3 (100.00)
Temporal lobe, Posterior lobe (*N* = 3)	2 (66.67)	0 (0.00)	0 (0.00)	0 (0.00)	0 (0.00)
Temporal lobe, Frontal lobe (*N* = 6)	4 (66.67)	0 (0.00)	1 (16.67)	1 (16.67)	0 (0.00)
Frontal lobe, Posterior lobe (*N* = 1)	0 (0.00)	1 (100.00)	0 (0.00)	0 (0.00)	0 (0.00)
Frontal lobe, Posterior lobe, Temporal lobe (*N* = 1)	1 (100.00)	0 (0.00)	0 (0.00)	0 (0.00)	0 (0.00)
Temporal lobe, Pons, Cerebellum (*N* = 1)	1 (100.00)	0 (0.00)	0 (0.00)	1 (100.00)	0 (0.00)
Frontal lobe, Temporal lobe, Parietal lobe, Posterior lobe (*N* = 1)	1 (100.00)	1 (100.00)	0 (0.00)	0 (0.00)	0 (0.00)
*p*	0.249	0.054	0.386	0.571	**0.015**

*p* for Fisher’s exact text; Significant difference is in bold.

**Table 6:** Prevalence of post-stroke outcome according to location of vascular changes.

Location of Vascular Changes	Post-Stroke Outcome				
	Hemiparesis	Epilepsy	Aphasia	Movement Disorders Other than Hemiparesis	Intellectual Delay
	*n* (%)	*n* (%)	*n* (%)	*n* (%)	*n* (%)
Total (*N* = 42)	27 (64.29)	4 (9.52)	6 (14.29)	3 (7.14)	7 (16.67)
MCA only (*N* = 14)	10 (71.43)	3 (21.43)	3 (21.43)	1 (7.14)	4 (28.57)
PCA only (*N* = 4)	1 (25.00)	0 (0.00)	0 (0.00)	1 (25.00)	0 (0.00)
ACA only (*N* = 3)	1 (33.33)	0 (0.00)	1 (33.33)	0 (0.00)	1 (33.33)
ICA only (*N* = 6)	3 (50.00)	0 (0.00)	0 (0.00)	1 (16.67)	0 (0.00)
ICA, ACA (*N* = 3)	3 (100.00)	0 (0.00)	1 (33.33)	0 (0.00)	1 (33.33)
ICA, MCA (*N* = 6)	6 (100.00)	0 (0.00)	1 (16.67)	0 (0.00)	1 (16.67)
ICA, ACA, MCA (*N* = 4)	2 (50.00)	0 (0.00)	0 (0.00)	0 (0.00)	0 (0.00)
MCA, ICA, PCA (*N* = 1)	0 (0.00)	1 (100.00)	0 (0.00)	0 (0.00)	0 (0.00)
ICA, MCA, PCA, ACA (*N* = 1)	1 (100.00)	0 (0.00)	0 (0.00)	0 (0.00)	0 (0.00)
*p*	**0.045**	0.157	0.552	0.809	0.429

ICA—internal carotid artery; MCA—middle cerebral artery; ACA—anterior cerebral artery; PCA—posterior cerebral artery. *p* for Fisher’s exact text; Significant difference is in bold.

## Data Availability

The data which we presented in the study are available from authors on request. The data are not publicly available due to privacy restrictions.
